# AI-Driven Aeronautical Ad Hoc Networks for 6G Wireless: Challenges, Opportunities, and the Road Ahead

**DOI:** 10.3390/s22103731

**Published:** 2022-05-13

**Authors:** Tuğçe Bilen, Berk Canberk, Vishal Sharma, Muhammad Fahim, Trung Q. Duong

**Affiliations:** 1 Department of Computer Engineering, Faculty of Computer and Informatics, Istanbul Technical University, Istanbul 34469, Turkey; bilent@itu.edu.tr; 2 Artificial Intelligence and Data Engineering Department, Faculty of Computer and Informatics, Istanbul Technical University, Istanbul 34469, Turkey; canberk@itu.edu.tr; 3 School of Electronics, Electrical Engineering and Computer Science, Queen’s University Belfast, Belfast BT7 1NN, UK; v.sharma@qub.ac.uk (V.S.); m.fahim@qub.ac.uk (M.F.)

**Keywords:** AANETs, AI-enabled networks, AI-driven AANETs, AANET management

## Abstract

Aeronautical ad hoc network (AANET) has been considered a promising candidate to complete the vision of “Internet in the sky” by supporting high-speed broadband connections on airplanes for 6G networks. However, the specific characteristics of AANET restrict the applicability of conventional topology and routing management algorithms. Here, these conventional methodologies reduce the packet delivery success of AANET with higher transfer delay. At that point, the artificial intelligence (AI)-driven solutions have been adapted to AANET to provide intelligent frameworks and architectures to cope with the high complexity. The AI-driven AANET can provide intelligent topology formation, sustainability, and routing management decisions in an automated fashion by considering its specific characteristics during the learning operations. More clearly, AI-driven AANETs support intelligent management architectures, overcoming conventional methodologies’ drawbacks. Although AI-based management approaches are widely used in terrestrial networks, there is a lack of a comprehensive study that supports AI-driven solutions for AANETs. To this end, this article explores the possible utilization of primary AI methodologies on the road to AI-driven AANET. Specifically, the article addresses unsupervised, supervised, and reinforcement learning as primary AI methodologies to enable intelligent AANET topology formation, sustainability, and routing management. Here, we identify the challenges and opportunities of these primary AI methodologies during the execution of AANET management. Furthermore, we discuss the critical issue of security in AANET before providing open issues.

## 1. Introduction

**Figure 1:**
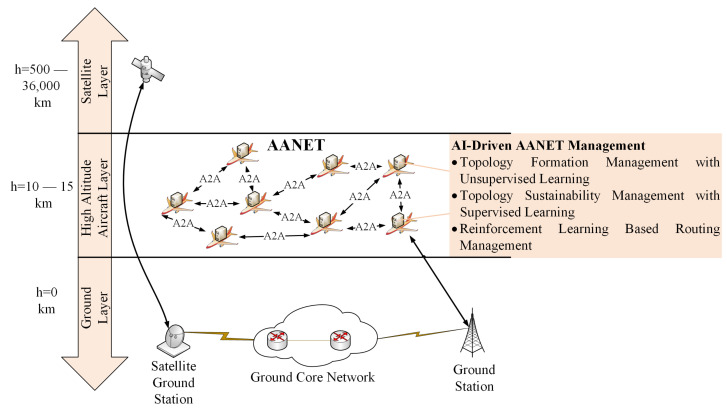
An exemplary illustration of AANET topology.

The satellite and air-to-ground (ATG) connectivity are the two pivotal methods to enable in-flight connectivity (IFC) for the next generation (6G) of mobile wireless networks [1]. However, the cost and coverage restrictions of these methods reduce the quality of the end-user experience. The aeronautical ad hoc networks (AANETs) can enable Internet connectivity to the aircraft that cannot directly access the terrestrial network, and accordingly, this extends IFC’s coverage area with cost reduction [2]. For this reason, AANETs have attracted a lot of attention from both industry and academia as a potential candidate to improve the coverage area and end-user experience of IFC with cost efficiency. These advantages are achieved by establishing air-to-air (A2A) links between airplanes that execute packet transfer, as shown in Figure 1. More specifically, airplanes’ packets are transferred through A2A links in an ad hoc manner until reaching the destination node having Internet connectivity [3,4]. Each plane acts as a router for packet transfer in this formed network without the assistance of any centralized terminal.

The AANET operation is to form a topology and sustain it to enable packet routing through A2A links during IFC. However, at this point, the use of existing topology formation, sustainability, and routing management algorithms is challenging due to the specific characteristics of AANETs [5]. Accordingly, the following section investigates the conventional solutions for topology formation, sustainability, and routing management challenges caused by the AANET-specific characteristics. Additionally, in this article, the specific characteristics of AANETs are considered as the high aircraft density in three-dimensional (3D) space, unstructured ad hoc topology, ultra-dynamic network, and packet transmissions through the A2A links. We also provide the details of these specific characteristics in Section 2.

### 1.1. Related Works

There are various literature works proposing clustering algorithms for AANET topology formation. A honeycomb division-based clustering algorithm to solve the scalability, cluster head bottleneck, and overlap problems presented in [6]. Here, the area is divided into hexagonal regions, and the head is selected from them by considering the overlap problem. The connectivity, throughput, and delay issues of aeronautical networks are considered in [7]. Here, they present the topology and investigate two main communication models: single-hop and two-hop. Finally, they aim to find an upper throughput bound for these communication models. The enhanced one-hop clustering algorithm is proposed to enable a stable clustering mechanism for AANETs [8]. The neighbor discovery, cluster head determination, and cluster merging are the primary considerations of this method. Two different clustering algorithms are proposed as Dynamic Doppler Velocity Clustering (DDVC) and Dynamic Link Duration Clustering (DLDC) in [9]. The DDVC is generally utilized for the pseudo-linear highly mobile large ad hoc networks. For this reason, it uses the relative velocity between the nodes obtained from the Doppler shift of control packets. On the other hand, the DLDC more accurately estimates the link expiration time by utilizing the nodes’ position and velocity parameters. In addition, as mentioned above, the main aim of aircraft clustering is to create more stable A2A links between airplanes. Therefore, link stability is an essential criterion for designing aircraft clusters. Due to the Doppler shift effect, the A2A link between the planes moving in opposite directions is unreliable; for this reason, the links are established between airplanes flying in the same direction [10,11]. The end-to-end delay related to AANET formation and resource allocation is aimed to reduce through solving integer nonlinear programming problems based on the realistic dynamic graph model in [12].

In addition to the above, few algorithms enable the sustainability of AANET in literature. Some works promote this sustainability through handover management similar to the cellular networks. The dual connectivity-based handover management algorithm is proposed with a software-defined networking controller on VHF and mobile user objective links in [13]. They aim to reduce the handover overhead by considering queue backlogs, user fairness, and limited resource constraints. A handover algorithm combining Mobile IP and Resource Reservation Protocols for aeronautical communications is proposed in [14]. The proposed handover algorithm consists of information collection, handover decision, and handover execution. The L-DACS 1 is integrated with realistic IP-based network layer functionality by analyzing the handover performance of this system [15]. This work triggers the handover procedure based on the signal level differences between neighbors. The energy efficiency, signal intensity, network cost, delay, and bandwidth are used to characterize the user preference and network performance through utility function definition [16]. Then, the authors use this function with multicriteria utility theory to design an energy-efficient network selection approach as a handover methodology. Finally, the handover algorithm to select a new Internet gateway that is the aircraft that is connected to the Internet is proposed in [17]. This algorithm makes the selection based on geographic proximity and a measure of Internet gateway congestion. Accordingly, the geographically closest and less loaded node for the handover procedure can be selected.

Moreover, numerous studies propose routing management algorithms for AANETs in the literature. A geographic routing protocol AeroRP is presented to enable multihop routing for highly dynamic aeronautical networks in [18]. The authors utilize velocity-based heuristics and decision metrics for the forwarding of packets. The security addition is implemented to AeroRP as SAeroRP in [19]. Here, the fake geographic location vulnerability is disabled by utilizing authentication and key transport mechanisms. The Ad-hoc Routing Protocol for Aeronautical Mobile Ad-Hoc Networks (ARPAM) as a multipurpose routing protocol for AANETs is proposed in [20]. The authors utilize the distance and number of hops between airplanes to select the shortest routing path. In addition to the distance vector, it could also be considered an on-demand routing protocol based on broadcasting messages. The greedy forwarding is utilized to route the packets to the nearest neighbour, and it also combines the position-based packet forwarding with joining the shortest queue principle [21]. The relative velocity and expected queuing delay of nodes are utilized to select the next node during routing according to the delay aware Multipath Doppler Routing (DMDR) [22]. In addition to the traffic control, they also utilize the Doppler value metric to enable stability during routing. There are various works based on these three management-level issues, and only some results among them take advantage of the AI-driven management in a limited way, as shown in Table 1.

### 1.2. Scope of Article and Main Contributions

As explained in the above section, there are various works in the literature proposing AANET topology formation, sustainability, and routing management algorithms. On the other hand, these algorithms cannot handle the AANET-specific characteristics simultaneously. Therefore, to overcome the AANET management challenges caused by its specific characteristics, we require intelligent frameworks and architectures specially designed for topology formation, sustainability, and routing procedures. This intelligence could be achieved by embedding artificial intelligence (AI)-based solutions to the design of AANETs. The embedding of AI to wireless networks is well accomplished [23,24]. However, the utilization of AI in AANET is an unexplored and relatively new research area.

In this article, we investigate the possible AI methodologies to ease the management of AANETs. More specifically, as shown in Figure 1, we will analyze the unsupervised, supervised, and reinforcement learning-based methodologies for AANET topology formation, sustainability, and routing management. Accordingly, we will investigate the AI-driven AANET management methodologies in three main categories. Additionally, we analyze the challenges and opportunities of these methodologies on the road ahead to AI-based AANETs. The main contributions of the paper could be summarized as follows:

- Examination of AI for AANET management: The utilization of AI-based methodologies for AANET management is still an unexplored area. This is the first work to investigate possible AI methodologies for AANET management.
- Investigation of 6G and AANET relation: This is the first work to highlight the importance of the relationship between core 6G supports and AANET-specific characteristics.
- Discovery of AANET’s specific characteristics: We describe AANET’s specific characteristics to highlight the necessity of AI for AANETs. These specific characteristics also become the starting point in AANET management problems.
- Discussion about AANET management problems: We discuss three main management problems for AANET caused by its specific characteristics.
- Matching of AI methodologies and AANET management problems: We give our specific attention to proposing AI-based methodologies in three main AANET management problems.

**Figure 2:**
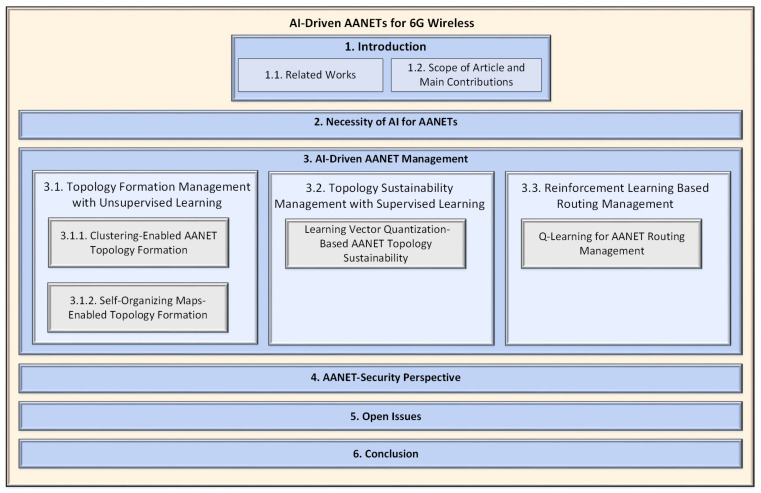
The general structure of article.

The rest of the paper is organized as follows: In Section 2, we explore the necessity of AI for AANET by discussing its specific characteristics. Section 3 investigates the AI-driven AANET management in terms of three main management-level challenges. The security issue of AANET is discussed in Section 4. The open issues and future directions are presented in Section 5. Finally, we conclude the paper in Section 6. Additionally, to increase the traceability of the article, we summarize the general architecture of it in Figure 2.

## 2. Necessity of AI for AANETs

Unlike other ad hoc networks, the AANETs have specific characteristics, which are the root causes for different management-level challenges. Before giving the details of these challenges, we first investigate the specific characteristics of AANETs by matching them with AI-based methodologies to underline the necessity of AI-based solutions.

- High Aircraft Density in 3D Space: One of the main characteristics of AANET is the movement of a large number of aircraft in the 3D environment, which produces a massive amount of data on heterogeneous characteristics (airplanes’ locations, speeds, and angles). Accordingly, this enormous amount of heterogeneous data overwhelms the management procedures of AANETs. AI has become the leading solution to learn and analyze these data for AANET management. Therefore, we can make estimations, create clusters, and recognize patterns in AANET by analyzing the collected information through AI.
- Unstructured Ad Hoc Topology: As the name suggests, the AANETs have an ad hoc characteristic that is independent of central guidance. Airplanes in the network can directly communicate with each other, and during this communication, each aircraft behaves as a router. Coupled with the ultra-dynamic network characteristic, this ad hoc infrastructure makes topology formation a challenging issue. Here, we can handle the topology formation of AANET through AI. At this point, we propose to utilize unsupervised learning due to its unlabeled data supporting character. Clearly, we can form a topology by trying to search for meaningful structures in an unstructured topology through unsupervised learning, as shown in Figure 3.
  **Figure 3:**
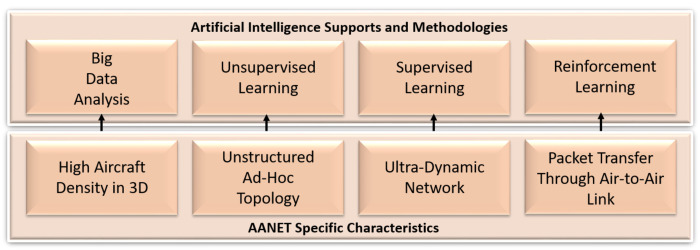
AI supports and methodologies for AANET-specific characteristics.
- Ultra-Dynamic Network: The ultra-dynamism is one of the essential AANET characteristics resulting from airplanes’ high speed. This over-speed makes the formed network ultra-dynamic by increasing the breakage rate of A2A links between airplanes. Accordingly, the AANET topology with aircraft nodes and A2A links changes continuously. However, continuous topology creation becomes time-consuming in this highly dynamic network. Instead, the initially formed topology could be utilized through supervised learning, as shown in Figure 3. To achieve this, we can consider the formed topology as training data for mapping the new instances by handling heterogeneity.
- Packet Transfer through A2A Link: The packets of the source aircraft are transferred through A2A links until reaching the destination to enable Internet connectivity. However, due to the ad hoc behavior and ultra-dynamic network characteristics, the determination of the next plane becomes challenging [25]. More specifically, the next plane should be determined dynamically according to the current situation of the network topology during packet transfer since the connected airplanes and links of an aircraft change continuously with their movements. Reinforcement learning could be one of the possible AI-based solutions to handle this dynamism by also increasing packet transfer success, as shown in Figure 3. Here, the airplanes can dynamically find their optimal packet transfer path through exploration and exploitation procedures without guidance.

**Figure 4:**
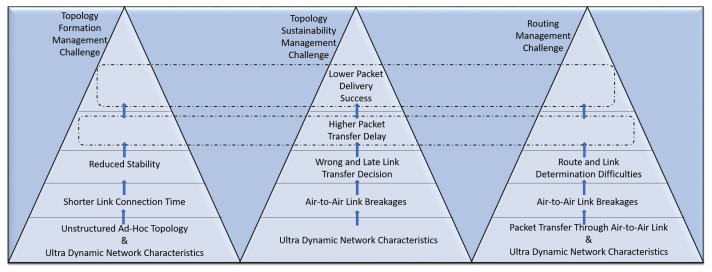
The root causes chains of AANET management challenges.

These specific characteristics become the origin of management level challenges, as illustrated in Figure 4. To overcome the AANET management challenges, we require AI-based intelligent frameworks and architectures. Therefore, they also underline the necessity of AI for AANETs. The following section of the article will detail each of these management-level challenges and suggest corresponding AI-driven solutions based on AANET’s specific characteristics.

Moreover, in literature, various works propose AANET algorithms without utilizing AI (i.e., [26,27,28]). However, these works only consider the general aspects of AANET instead of focusing on its management. Additionally, these works do not consider the effects of the AANET-specific characteristics simultaneously. On the other hand, we should handle these AANET-specific characteristics simultaneously to manage the challenging AANET environment. At that point, we should utilize AI-based solutions to overcome the problems observed during AANET management caused by its specific characteristics.

## 3. AI-Driven AANET Management

One of the crucial needs of 6G is to enable seamless connectivity for harsh environments, and Internet connectivity should be easily sustained in all circumstances and conditions. With this connectivity support, 6G can gain an advantage over other generations. At that point, airplanes are one of the main harsh areas of Internet connectivity. Although the satellite and air-to-ground networks enable Internet connectivity to airplanes, their high cost and low coverage problems reduce efficiency. Here, the AANETs can enable Internet connectivity to airplanes by extending coverage areas with cost reduction. For this reason, the AANETs will be very important for the next-generation 6G concept. Moreover, the core features of 6G support the AANET by enabling support to its specific characteristics, as given in Section 2. Therefore, there is a strong relationship between the core 6G supports and AANET-specific characteristics. We summarize the 6G and AANET relation by matching these supports and characteristics in Table 2.

As summarized in Table 2, the higher data rate of the 6G supports the enormous aircraft density of AANETs in 3D place. As explained in Section 2, the AANETs produce a massive amount of data with heterogeneous characteristics. Here, 6G enables support to effectively manage this enormous amount of heterogeneous data with a higher data rate. Additionally, the continuous topology creation increases the latency in the ultra-dynamic AANET, and the lower latency of 6G enables a solution to this time-consuming operation, as summarized in Table 2. Additionally, the higher reliability and accuracy of 6G enable the effective formation of unstructured AANET topology. Similarly, the routing of packets on AANET is challenging due to unstable A2A link characteristics. 6G can execute this transfer in an energy-efficient manner thanks to its terahertz (THz) bands. Finally, to support our proposed AI-driven AANET management, 6G becomes one of the critical solutions due to its AI-supportive features. After explaining the importance of the 6G and AANET relation, we aim to enable an insight into AI-driven solutions for the main AANET management challenges, as detailed in the rest of the article.

In the previous section, we explained the necessity of AI for AANETs by matching the critical AI methodologies with AANET-specific characteristics. In what follows, we investigate the details of three management challenges caused by AANET’s specific characteristics. We aim to handle each of these management challenges with AI-based methodologies. At that point, the AI-driven methodologies enable fast adaptation of airplanes to the dynamic conditions of AANETs [29]. More specifically, the aircraft can form and sustain the AANET topology by finding routes according to the instant status of AANETs. In this way, the dynamic conditions of AANETs could be learned by airplanes thanks to AI-driven methodologies. The aircraft could then make the management decisions dynamically without any guidance. As explained in the previous section, we divide the management issues of AANETs into three main categories: topology formation, topology sustainability, and routing. In the rest of the article, we aim to investigate and propose corresponding AI-driven methodologies for these management challenges.

### 3.1. Topology Formation Management with Unsupervised Learning

As explained in Section 2, the unstructured ad hoc topology is one of the main AANET characteristics. This infrastructure complicates the formation of AANET topology when combined with ultra dynamism. These two characteristics reduce the stability of the AANET topology by shortening the connection life of the links between two airplanes. This unstable network with short-lived links deteriorates the packet delivery success by increasing transfer delay, as illustrated in Figure 4. We can handle the unstructured and ultra-dynamic characteristics of AANETs if we support topology formation management with AI. Accordingly, we can improve the packet delivery success with a shorter delay.

Unsupervised learning is the most relevant AI concept for AANET topology formation management. Here, the main aim of unsupervised learning is to find previously unknown patterns in data. Accordingly, it aims to explore the data to find meaningful structures and define classes. If we think about AANETs, we have independently located airplanes with unstructured characteristics before forming the topology. They could be considered as the unlabeled training data for unsupervised learning. Accordingly, our main aim is to group the airplanes with similar features under one cluster to form the AANET topology [30]. Therefore, we will investigate the role of two main unsupervised learning methods in topology formation: clustering and self-organizing maps.

#### 3.1.1. Clustering-Enabled AANET Topology Formation

**Figure 5:**
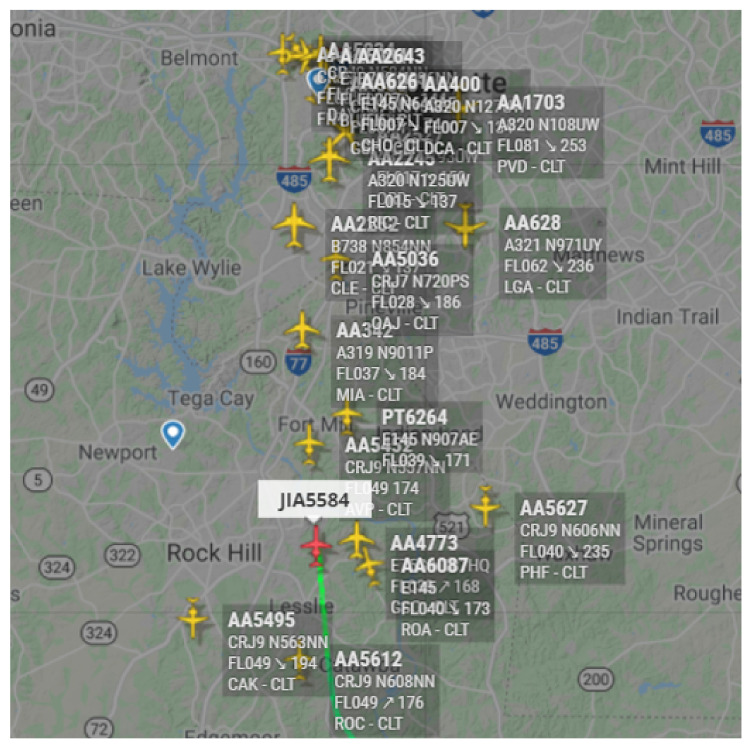
Exemplary aircraft cluster on instant real traffic (from 1 December 2021 flightradar24.com) [32].

The main aim of the clustering-enabled AANET topology formation is to create different aircraft groups according to their similarities [31]. In AANETs, we utilize the clustering for grouping airplanes according to their similarities in direction, distance, angle, altitude, and mobility characteristics as shown in Figure 5 based on Flightradar24 databases [32]. Therefore, the airplanes in the same cluster can establish more long-lasting A2A links to increase the stability of AANET. In this article, we investigate three clustering methods that are mainly utilized for AANETs in terms of their advantages and disadvantages, as explained below:

- Prototype-based Clustering: It assigns the instances to the nearest prototypes, and the K-means clustering constitutes one of the primary examples. It can easily be implemented and effectively work with large data sets. This enables high-speed performance for the ultra-dense and 3D AANET environment. However, the cluster number should be assigned before the algorithm runs, which contradicts the ultra-dynamic characteristic of AANET as summarized in Table 3. Here, the K-means is one of the primary examples of this type of clustering as explained above, and its complexity is the *O*(*n*^2^).
- Hierarchical Clustering: The hierarchical clustering aims to create clusters based on the previously established ones. During this creation, two different algorithms, agglomerative and divisive, could be utilized. The divisive approach starts with one initial cluster and then splits this into child clusters. Conversely, the agglomerative clustering starts from smaller groups to form larger clusters according to the bottom-up approach. In this clustering, we do not specify the number of clusters initially, which is the main advantage of the dynamic environment of AANETs, as summarized in Table 3. On the other hand, the algorithm does not return from the previously completed operation, which contradicts the unstructured ad hoc topology of AANETs since the clusters need to be updated according to the formed topologies. Additionally, the complexity of the hierarchical clustering is *O*(*kn*^2^), as presented in Table 4. Here, *n* represents the clustered elements while *k* becomes the number of clusters.
- Density-based Clustering: Density-based clustering can discover non-linear clusters with arbitrary shapes. This feature increases the clustering performance in the unstructured topology of AANETs. Similar to hierarchical clustering, this method also does not require the defined cluster number [33]. However, the AANETs have high and changing aircraft density, while the algorithm does not efficiently work on the varying density clusters, as summarized in Table 3. Here, as shown on Table 4, the complexity of the density-based clustering corresponds to the *O*(*nlogn*).

#### 3.1.2. Self-Organizing Maps-Enabled Topology Formation

Clustering is mainly utilized to form the AANET topology, as explained above. However, other unsupervised learning methodologies could also be used for AANET topology formation. Here, we introduce the Self-Organizing Maps (SOMs), an artificial neural network based on unsupervised learning. The SOMs use competitive learning to cluster the distinct patterns from the input data into output sets. SOMs can handle the previously unknown inputs, and this strongly matches the ultra-dynamic characteristic of the AANETs, as summarized in Table 3. Therefore, SOMs could be utilized to create the unstructured AANET topology in the form of clusters. The clustering performance of the SOMs is not affected by the unstructured AANET environment since it keeps the relative distances steady in a cluster. Accordingly, the SOMs can handle the newly included aircraft to the AANET for cluster assignment.

After explaining the main reasons for selecting the SOMs for topology formation, we aim to adapt it for AANETs. During this adaptation, each aircraft in AANET corresponds to the input vector in SOMs. These input nodes are directly connected to the output without any hidden layer, and here, each connection is associated with a weight value. In the output layer, the AANET clusters become the output vectors, and the number of these represents the maximum cluster number of the AANET [34]. These clusters are connected through intra-layer links without weight values. Additionally, in the output layer, the topologies could be mapped in the form of a cylinder or toroid instead of the hexagonal structure due to the 3D environment of AANETs. Additionally, the Euclidean distances could be utilized by determining the similarities between input vectors to execute this mapping. Thanks to these features, we can map the 3D space of the AANET onto a plane with SOMs by preserving the underlying structure. However, determining the optimal map size is already challenging in SOMs. This decision will be more difficult for AANET due to the high and changing aircraft density in the 3D environment, as summarized in Table 3. Additionally, the complexity of this algorithm becomes *O*(*n*^2^), as noted in Table 4. Here, *n* represents the size of the samples.

**Table 3:** AANET Related Opportunities and Challenges of AI-Driven Methods.

Methods	Advantages for AANET	Disadvantages for AANET
Prototype-based Clustering	⋄ Effectively works for large data sets ⋄ Supports high aircraft density in 3D	⋄ Previously defined cluster number ⋄ Contradicts ultra-dynamic network
Hierarchical Clustering	⋄ Undefined cluster number ⋄ Supports ultra-dynamic network	⋄ Unchanged priori operation ⋄ Contradicts unstructured topology
Density-based Clustering	⋄ Undefined cluster number ⋄ Supports ultra-dynamic network ⋄ Nonlinear clusters with arbitrary shape ⋄ Supports unstructured ad hoc topology	⋄ Failure on the varying density clusters ⋄ Contradicts high aircraft density in 3D
Self-Organizing Maps	⋄ Topology protection without changing relative distances ⋄ Supports unstructured ad hoc topology ⋄ Processing of previously unknown inputs ⋄ Supports ultra-dynamic network	⋄ Difficulty on optimal map size decision ⋄ Contradicts high aircraft density in 3D
Learning Vector Quantization	⋄ Efficient pattern classification ⋄ Supports ultra-dynamic network ⋄ Efficiency for multiclass classification ⋄ Supports unstructured ad hoc topology	⋄ Lower success on high-dimensional data ⋄ Contradicts high aircraft density in 3D
Q-Learning	⋄ The ability to work in changing areas ⋄ Supports ultra-dynamic network ⋄ Sequential decision making ⋄ Supports transfer through A2A link	⋄ High-dimensional state and action space ⋄ Contradicts high aircraft density in 3D

### 3.2. Topology Sustainability Management with Supervised Learning

The ultra-dynamic network constitutes one of the main characteristics of the AANET caused by the high speed of airplanes. As detailed in Section 3.1, this ultra-dynamism complicates the formation of a stable topology. The sustainability of this formed topology also becomes challenging due to ultra-dynamism. More specifically, the formed topology is continuously disrupted due to the breakages of A2A links between airplanes [35]. On the other hand, to enable the sustainability of the AANET topology, the broken links should be established again with different airplanes. Here, the wrong and late link transfer decisions again reduce the packet delivery success with higher delay, as illustrated in Figure 4. These effects make the sustainability management of an AANET challenging. Unlike the works described in Section 1.1, we can utilize the initially formed topology to enable the sustainability of AANET instead of creating it continuously.

At that point, we can take advantage of *Supervised learning* to ensure the sustainability of AANET based on the initial topology. More specifically, we can use the initially formed topology as a training set to determine its upcoming states due to supervised learning can produce output by utilizing previous experience [36,37]. Moreover, thanks to the classification category of supervised learning, we can facilitate the assignments of new airplanes to clusters identified with unsupervised learning. Based on these arguments, in the following parts, we will exemplify a supervised learning method for AANET topology sustainability as learning vector quantization by also giving its opportunities and challenges during this operation.

#### Learning Vector Quantization-Based AANET Topology Sustainability

The learning vector quantization (LVQ) could be considered the supervised version of the SOM. Similarly, the LVQ classifies the patterns into output units that denote classes. More clearly, the initially given network and output classes are utilized to classify an input vector [38]. This working principle strongly matches the topology sustainability requirement of ultra-dynamic AANETs. Therefore, we can enable the sustainability of the initially formed AANET topology by using the pattern classification feature of this methodology since it is more effective to ensure the sustainability of ultra-dynamic AANET topology rather than reforming it continuously. Additionally, it can be easily implemented for multiclass classification problems, which increases the efficiency of grouping in the unstructured environment of AANETs. Accordingly, it allows for the separate grouping of airplanes with different characteristics. The newly included aircraft could also be placed in a previously formed suitable group of AANET without reclustering thanks to LVQ.

Therefore, we can utilize the LVQ for topology sustainability management in AANETs. Accordingly, our main aim is to find the best matching cluster for an aircraft observing A2A link breakage [39]. Here, we take advantage of this AI-based approach to prevent continuous topology formation. Instead, we utilize the initially formed topology in pattern classification by utilizing the aircraft clusters in the formed AANET topology as *pre-classified training data* in *multidimensional space* of AANET. Here, the *weight vectors* are the airplanes in the formed AANET cluster. Additionally, the aircraft with a broken A2A link corresponds to the *input vector*. Accordingly, each known aircraft cluster becomes *output*. We aim to sustain the AANET topology instead of continuous topology formation by utilizing LVQ. Although LVQ is critical for enabling topology sustainability, it can achieve lower success on high-dimensional data. This situation contradicts the high aircraft density characteristic of AANETs in 3D, as summarized in Table 3. The high aircraft density of AANETs increases the computational cost of applying LVQ [40]. Here, we can also observe the same complexity with the self-organizing maps.

**Table 4:** Complexities of AI-Driven Methods.

Methods	Complexities
Prototype-based Clustering	*O*(*n*^2^)
Hierarchical Clustering	*O*(*kn*^2^)
Density-Based Clustering	*O*(*nlogn*)
Self-Organizing Maps	*O*(*n*^2^)
Learning Vector Quantization	*O*(*n*^2^)
Q-Learning	*O*(*n*^3^)

### 3.3. Reinforcement Learning-Based Routing Management

As explained in Section 2, the AANETs work based on the principle of packet transfer over A2A links. The routing management creates an inevitable need to deliver the packets of source aircraft to the destination by determining A2A links. On the other hand, the routing protocols used in terrestrial networks do not satisfy the requirements of AANETs due to ultra-dynamism. More specifically, ultra-dynamic characteristics of AANET increase the breakage rates of A2A links between airplanes, as explained in Section 2. This situation also makes the route and link determination difficult during routing, as illustrated in Figure 4. The difficulty in making a route decision reduces the packet delivery success with higher delay. Therefore, routing management in AANET becomes another challenging issue.

Unlike the routing algorithms given in Section 1.1, reinforcement learning enables us to make routing decisions in the ultra-dynamic environment of AANET by also maximizing packet delivery success [41]. Clearly, reinforcement learning makes immediate routing decisions by exploring A2A links through experience [42]. Thus, each aircraft can sequentially determine its routing path with reinforcement learning in two different ways: model-based and model-free. The model-based reinforcement learning makes decisions based on the model of the environment, and it is not suitable for the AANETs due to its dynamic characteristic. On the other hand, model-free reinforcement learning can only make decisions with instant exploration without any environment model, which makes it very applicable for AANETs. For this reason, we will examine the implementation details of Q-learning, one of the main model-free reinforcement learning techniques, in the upcoming section.

#### Q-Learning for AANET Routing Management

**Figure 6:**
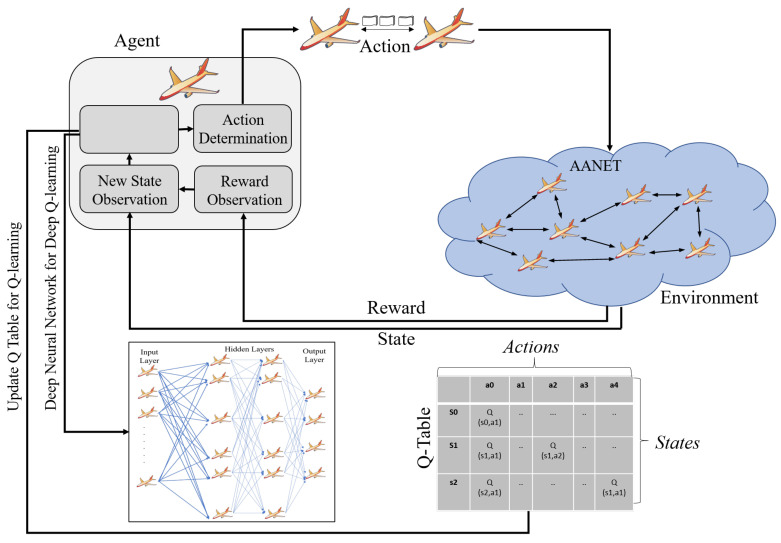
Q-Learning and Deep Q-Learning for AANETs.

Q-learning is a model-free reinforcement learning method working based on action and reward mechanism [43]. This flexibility makes it easy to adapt to the dynamic and unstructured AANET environment. Here, the aircraft can explore different A2A links to choose the one with high packet delivery success as a reward. Clearly, the Q-learning considers the dynamism of the AANET environment, and the previous decisions affect the subsequent actions [44]. Accordingly, this sequential decision making facilitates path selection during routing with higher packet rates. Therefore, in Q-Learning, we assume each aircraft as an agent with a state, then this agent takes action, earns a reward, and switches to a new state, as shown in Figure 6. More clearly, Figure 6 summarizes the mapping of Q-learning concepts to the AANET scenario. In this mapping, the AANET creates the *Environment* of Q-learning, and each aircraft performing packet transfer through A2A link in this environment becomes an *Agent*, as shown in Figure 6. Accordingly, the packet transfers executed through the A2A links could be defined as an action. These actions are included by the action set changing according to the connections of airplanes, as shown in Figure 6. Based on these, the delivery success of the A2A packet transfer denotes the *Reward *(*R*). Therefore, a reward is obtained according to the result of each action. The obtained rewards are used to fill the *Reward Table*. Here, each cluster in an AANET is represented with a reward table. This table shows the airplanes in that cluster with rows and columns. Accordingly, the rewards for the corresponding links are placed in the cells of this table. After obtaining rewards and filling the reward table, we can observe the new states, as shown in Figure 6. The *State *(*s*) represents the status of an aircraft according to its packet transfers. These determined states and rewards help us to update the Q-Table that enables aircraft to learn the environment as a result of experiences. Then, each aircraft can determine the actions in the next step. At this point, the Bellman Equation, as given with Equation (1), is utilized to fill the Q-table.

(1) $$\begin{array}{c} Q_{t}\left(s,a\right)=Q_{t-1}\left(s,a\right)+\alpha (R\left(s,a\right)\\ +\gamma \max_{a^{\prime }}Q\left(s^{\prime },a^{\prime }\right)-Q_{t-1}\left(s,a\right)) \end{array}$$

According to the Q-learning concept, aircraft can choose the path that maximizes Equation (1) by considering all possible actions that can happen for all neighbors. In Equation (1), *s* shows the current state of an aircraft while *a* denotes an action executed in that state. The *R*(*s*,*a*) is a reward value obtained by taking action *a* in state *s*. The *Q*(*s*) equals the value of being in a particular state, and the *s*′ is the state coming after *s* by taking action *a*. Finally, *γ* is defined as a discount factor. Additionally, the complexity of this algorithm is the *O*(*n*^2^), as summarized in Table 2. Here, n represents the size of the state space. Although Q-learning is directly applicable to dynamic environments, its high-dimensional state and action space can increase the computational load when combined with the high aircraft density of AANETs in 3D, as summarized in Table 3. More specifically, the memory and time requirement of the algorithm is increased with a growing Q-table. At that point, the *Deep Q-Learning* could be utilized to solve the performance defects caused by the complex action, and state-space [45,46]. In deep Q-learning, the neural network is used to approximate the Q-value function instead of table usage, as shown in Figure 6. The neural network outputs the Q-value of all possible actions when it receives the initial value. Therefore, deep Q learning can become a more scalable solution for the environments that require more complex action and state schemes [47].

There are various works based on these three management-level issues, and only some results among them take advantage of the AI-driven management in a limited way, as shown in Table 1. Therefore, as a solution to these conventional methodologies, in this article, we aim to solve three main management problems on AANETs by utilizing AI-based methodologies. In the first step, we should create the AANET topology in the form of clusters. Then, we should enable its sustainability, and finally, we can route the packet from the source aircraft to the destination. Accordingly, this article aims to solve these management issues through the unsupervised, supervised, and reinforcement learning methodologies. We only have airplanes without any labels and clusters at the first step. For this reason, we should process them to create the AANET in the form of clusters, and we can achieve this through unsupervised learning. Then, in the second step, we have labeled and clustered the AANET topology, and we can enable its continuity through supervised learning. Finally, we should route the packets to the destination with corresponding algorithms. We can choose different AI-based methodologies from the unsupervised, supervised, and reinforcement learning categories at each step. This article only selects some important and applicable algorithms from these categories in each step.

In addition to the AANET A2A links with the above-explained characteristics, different connection types could be used for various use cases. We summarize the important connection types and the related use cases in Table 5.

## 4. AANET—Security Perspective

Security is hugely related to the mode in which the networks operate. With ad hoc features, the security needs to be swift and flexible. In the case of AANETs, transmission security becomes an ultra problematic area as an attack on the AANETs system can be devastating, leading to huge losses. Moving towards the pillars of confidentiality, integrity, authentication, and availability, security will become complex given the topology adjustment because of the increasing density of aircraft. The attackers may exploit the sensitive equipment on board, which may severely impact flight monitoring, fabricated path generations, navigation, and data sharing. One of the aims of the AANETs is to provide distributed services across the passengers through high-speed channel facilitation, which would require addressing traditional security issues associated with ad hoc formations such as synchronization, exposed terminals, resource reservation, and adaptive rate control. It would be an additional challenge to the security to facilitate location-dependent contention when data is transmitted across airplanes. An incorrectly configured security protocol can increase route acquisition delays, making the shared channel prone to errors. Adversaries can compromise the lookup tables to initiate loops in data transmission and impact the scalability of operations. Fast, flexible, and adaptable encryption is required when dealing with security provisioning in a highly dynamic setup, such as AANETs. Physical layer security and throughout maximization are challenges to be resolved in AANETs that can be handled through multiple access schemes [48]. As such, physical layer security can be an efficient tool to cope with the increased concern in secured AANETs [49,50].

Another challenge is that commercial flights are traceable, which means that it also reveals the coordinates, and each flight in itself is a terminal. Hence, location-based attacks would be prominent that need careful addressing and resolution. A loop attack on the lookup tables would cause a denial of service, further affected by the age of information, as the topology is expected to change rapidly, and information loss would be very high. When dealing with AI for AANETs, securely configuring the algorithms would be a challenge as it will have a tradeoff of performance security associated with it, which must not be compromised towards performance without balancing the security. Here, deep learning could be a go-to area for maximizing the secrecy rate [51]. Additional challenges would involve host identification and content revelation, as the passenger information can be traced based on the location-based services—this would be an interesting area to explore from the privacy point of view in AANETs. Additionally, existing threats related to buffer overflow, host impersonation, information disclosure, and interference would severely impact the functionality of AANETs. These threats and attack possibilities require further investigation considering the difference in links used to facilitate data transmissions, such as the satellite-assisted ad hoc formation or ground-assisted ad hoc formations [52,53,54]. Furthermore, provisioning cloud services across the AANETs and securing them with terrestrial support would be an area to explore and investigate. Facilitating such services from high-speed terrestrial networks would require security equilibrium with the dynamic topology of AANETs.

## 5. Open Issues

In this article, we have shown that the AANET’s specific characteristics mainly cause the management challenges of AANETs, and these challenges could be solved by utilizing different AI methodologies. During these investigations, we considered four AANET-specific characteristics as the origin of management challenges. However, the other characteristics of AANETs caused by altitudes, such as oxygen absorption, rain attenuation, and cloud attenuation, could be considered since they can lead to various management challenges by decreasing the ATA link qualities.

In addition to these management-level challenges, the AANET can experience different problems in terms of the data link and medium access control (MAC) layers due to its specific characteristics. Generally, these problems gather around link establishment, stability, and connectivity issues. The direction, free space loss, transmission range, communication range, and distance could be the main parameters for these issues. We can also utilize different AI methodologies to adjust these parameters automatically.

## 6. Conclusions

The AANET will be of great importance for 6G networks because it can enable seamless connectivity to one of the main harsh environments. In this article, we propose using AI-based methodologies to overcome the AANET management challenges caused by its specific characteristics. The article investigates these management challenges in three main aspects: topology formation, sustainability, and routing management. Accordingly, the AI-based methodologies enable us to obtain intelligent frameworks and architectures for these management issues. For this aim, we first underline the necessity of AI by matching its primary methodologies with AANET-specific characteristics. Here, we considered unsupervised, supervised, and reinforcement learning as primary methodologies for these management challenges on the road ahead to AI-driven AANET. We reviewed the challenges and opportunities of different approaches from these methodologies to automatically make topology formation, sustainability, and routing management decisions on AANET. Correspondingly, we investigated clustering and self-organized maps as unsupervised learning methodologies, while the learning vector quantization was analyzed under supervised learning. Finally, we evaluated the reinforcement learning to make routing decisions on AANETs. As a result of these, we aim to provide an insight into the road ahead towards AI-based AANET management.

## Figures and Tables

**Table 1:** Summary of Works on AANET Management.

Ref.	Management Concept	Methodology	AI-Addition	Parameters
[9]	Topology formation	Link Stability	Limited	Relative Velocity Link Expiration Time
[10]	Topology formation	Link Stability	None	Per-node Capacity Network Traffic
[11]	Topology formation	Link Stability	None	MAC Protocol
[12]	Topology formation	Resource Allocation	None	Dynamic Graph Model
[13]	Topology sustainability	Handover	None	Queue Backlog User Fairness Resource Constraints
[14]	Topology sustainability	Handover	None	PATH Messages
[15]	Topology sustainability	Handover	None	Signal Intensity
[16]	Topology sustainability	Handover	None	Utility Function (Delay, Bandwidth, etc.)
[17]	Topology sustainability	Handover	None	Geographic Proximity Congestion
[18]	Routing	Geographical Routing	None	Velocity-Based Heuristics Decision Metrics
[19]	Routing	Secure Geographical Routing	None	Velocity-Based Heuristics Decision Metrics Security Protocols
[20]	Routing	Distance Vector Routing On-Demand Routing	None	Broadcast Messages Distance Hop Count
[21]	Routing	Multipath Routing Geographic Routing	None	Speed of Advance of Packet
[22]	Routing	Reactive Routing Multipath Routing	None	Doppler Value Queuing Delay Relative Velocity

**Table 2:** Related Features of 6G and AANETs.

Core 6G Supports	AANET-Specific Characteristics
Higher Data Rate	High Aircraft Density in 3D Space
Lower Latency	Ultra-Dynamic Network
Network Reliability And Accuracy	Unstructured Ad Hoc Topology
Energy Efficiency	Packet Transfer through A2A Link
AI Support	AI-Driven AANET Management

**Table 5:** Currently utilized use cases and connection types for aircraft communications.

Use Case	Connection Type	Requirement
Backbone Connections Flight Tracking	Optical Links	Higher reliability
Simultaneous Data Transmissions	ATG with LTE (multi-user beamforming)	Higher spectrum utilization
Real-time video	Satellite connectivity	Higher bandwidth Lower delay Lower loss tolerance
Texting	ATG with LTE	Lower Bandwidth
Web browsing	ATG with LTE	Lower loss tolerance
Mail	ATG with LTE	Lower bandwidth
Video Streaming	Satellite connectivity	Lower delay Lower jitter Higher bandwidth
